# Fox River Valley Medical Association

**Published:** 1867-08

**Authors:** S. B. Hawley

**Affiliations:** Secretary *pro tem.*


### FOX RIVER VALLEY MEDICAL ASSOCIATION.

The regular quarterly session was held at Turner Junction, DuPage County, Ill., July 1st, 1867. Present—Dr. Winchester, President of the Association, and Drs. Jassoy, Young, Hawley, Patterson, LeBaron, Beggs, Crabtree, Tefft, Burbank, Goodwin, Olson, and Tyler.

The meeting was called to order by the President. Dr. Hawley was elected Secretary *pro tem.*

The minutes of the last meeting were read and approved.

On motion of Dr. Young, Dr. Tyler, of Massachusetts, was invited to a seat with the Association, and to join in the discussions.

Dr. Young proposed Dr. R. J. Patterson, of Batavia, as a member of the Association. He was elected, and with Drs. Beggs, Olson, and Burbanks, signed the Constitution and By-Laws, and paid the fees which constitute them full members.

Dr. Hawley, Chairman of Committee on Revision of Constitution and By-Laws, reported progress, and asked for further time in behalf of the Committee, which was granted.

Dr. Patterson presented a written communication, containing some hints on giving testimony in Courts, in cases of monomania and moral insanity. His large experience among the insane added much to the interest with which the paper was received, and to the interest of the general discussion of the subject which followed, by most of the members present.

Dr. Jassey moved a vote of thanks to the Doctor for his communication, and that he be invited to continue the subject at future meetings, which motion was unanimously carried.

Dr. Burbank reported a case of hepatic abscess, in which there had been a discharge of about fifty gall stones, of the size of a half-pea, and under. These stones showed, upon analysis, a composition of cholestrine, mostly. Result, recovery.

Dr. Goodwin reported a case of insanity, attended by many and long continued delusions on some points. A *post mortem* showed the organic disease of the patient to be mainly in connection with the stomach and liver.

Dr. Olson reported a case of paralysis, in an aged person, attended with a singular manifestation of external heat for many days before death.

Dr. Young reported a case of fracture of the humerus in a lady 84 years of age, in which perfect unison was obtaiued.

Dr. Hawley reported a case of compound comminuted fracture of the tibia, and recovery without suppuration. Patient aged 55, Also, a couple of cases of bronchitis in persons of a consumptive tendency, which were of interest mainly from the marked improvement which followed a change of treatment from neauseant and anodyne to stimulant and anodyne.

Dr. Young called attention to some recent observations of Drs. Davis and Johnson, tending to prove that alcoholic medicines lowered the force and frequency of the pulse, and also the temperature of the body, which called forth considerable discussion upon alcohol in general.

Dr. Beggs was named as Essayist at the next meeting, which takes place the first Monday in October, at Turner Junction.

On motion of Dr. Burbank, Association adjourned till the above date. S. B. HAWLEY, M.D., Secretary *pro tem.*

